# Effects of Exposure to Road, Railway, Airport and Recreational Noise on Blood Pressure and Hypertension

**DOI:** 10.3390/ijerph18179145

**Published:** 2021-08-30

**Authors:** Davide Petri, Gaetano Licitra, Maria Angela Vigotti, Luca Fredianelli

**Affiliations:** 1 Department of Biology, University of Pisa, 56126 Pisa, Italy; davide.petri@unipi.it (D.P.); mariangelavigotti@gmail.com (M.A.V.); 2 Institute for Chemical-Physical Processes, National Research Council, 56124 Pisa, Italy; 3 Clinical Physiology Institute, National Research Council, 56124 Pisa, Italy; 4 IPool S.r.l., Via Cocchi 7, 56121 Pisa, Italy

**Keywords:** noise, hypertension, environmental noise, railway noise, recreational noise, airport noise, road traffic noise, blood pressure, noise annoyance, diastolic blood pressure

## Abstract

Noise is one of the most diffused environmental stressors affecting modern life. As such, the scientific community is committed to studying the main emission and transmission mechanisms aiming at reducing citizens’ exposure, but is also actively studying the effects that noise has on health. However, scientific literature lacks data on multiple sources of noise and cardiovascular outcomes. The present cross-sectional study aims to evaluate the impact that different types of noise source (road, railway, airport and recreational) in an urban context have on blood pressure variations and hypertension. 517 citizens of Pisa, Italy, were subjected to a structured questionnaire and five measures of blood pressure in one day. Participants were living in the same building for at least 5 years, were aged from 37 to 72 years old and were exposed to one or more noise sources among air traffic, road traffic, railway and recreational noise. Logistic and multivariate linear regression models have been applied in order to assess the association between exposures and health outcomes. The analyses showed that prevalence of high levels of diastolic blood pressure (DBP) is consistent with an increase of 5 dB (A) of night-time noise (β = 0.50 95% CI: 0.18–0.81). Furthermore, increased DBP is also positively associated with more noise sensitive subjects, older than 65 years old, without domestic noise protection, or who never close windows. Among the various noise sources, railway noise was found to be the most associated with DBP (β = 0.68; 95% CI: −1.36, 2.72). The obtained relation between DBP and night-time noise levels reinforces current knowledge.

## 1. Introduction

Noise pollution represents a great public concern. Long-term exposure to high noise levels (>85 dB) have been associated with many direct health effects, even leading to hearing loss [1,2], or to non-hearing effects when exposure is at low-medium levels [3,4]. In this case, transportation noise can induce annoyance [5,6,7,8], sleep disturbance with awakening [9,10], cognitive impairment [11,12,13], physiological stress reactions [14], endocrine imbalance and cardiovascular disorders [15,16,17,18]. Moreover, exposure to noise can reduce both workers’ and students’ performance [19,20,21]. Higher levels of stress among subjects exposed to noise level higher than 55 dB (A) and increased occurrence of cardiovascular diseases associated with noise level greater than 65 dB (A) have also been reported [22]. Most of all, hypertension is the leading risk factor for cardiovascular morbidity and mortality worldwide [23]. Indeed, hypertension is a major risk factor for premature death and disability from heart disease, stroke, peripheral vascular disease and kidney failure [24,25,26]. A meta-analysis [24] evidenced a significant rise in prevalence of hypertension per increase of 5 dB (A) of equivalent road traffic noise level A weighted over a 16 h period (L_Aeq,16h_) (Odds Ratio (OR) = 1.03; 95% confidence interval (CI): 1.01–1.06). Moreover, results on the association between long-term exposure to noise and blood pressure (BP) are still heterogeneous [27,28,29,30]. A possible explanation was provided by Babisch [31,32,33], who suggested that an increase in the level of adrenaline, noradrenaline and cortisol in response to noise-induced stress could result in peripheral vasoconstriction, increased heart rate and a rise in arterial blood pressure. A lack of data on multiple sources of noise and cardiovascular outcomes is still an issue in the scientific literature.

Health impact assessment studies estimated that 104 million U.S. citizens have sufficient annual noise exposure to be at risk of noise-related health effects [34]. In Europe, even 15 years after the implementation of the Environmental Noise Directive [35], 40% of the European population remains exposed to road traffic noise levels over 55 dB (A) of L_den_ (average noise level over a 24 h period) and 15% to levels greater than 65 dB (A). Road traffic remains the most widespread source in the urban environment, followed by railway noise, with 22 million people exposed to noise levels higher than 55 dB (A) of L_den_ [36], then by aircraft noise with more than 4 million people, and industrial noise with 1 million people exposed. The scientific community has studied how different sources generate noise and how to mitigate this with innovative solutions, especially in an urban context with its main sources being road traffic [37,38], railway traffic [39], airport [40,41], industries [42,43] and port activities [44,45], where present. 

The impact of air traffic noise is particularly relevant during take-off and landing phases [46,47] if ground taxing operations are incorrectly managed [48]. Specific studies have been dedicated to aircraft noise’s relation with sleep disturbances and annoyance [49,50,51], while others, including the HYENA and SERA projects, focused on blood pressure and the risk of hypertension [46,52,53,54]. The HYENA project aimed at assessing the impacts on cardiovascular health of noise generated by air traffic and road traffic near six European airports. The results showed significant exposure–response association between night-time aircraft noise, daily road traffic noise and prevalence of “heart disease and stroke” and hypertension

Railways received specific attention in the ALPNAP study [55,56], where significant associations between railway noise and sleep medication intake were shown, especially for people exposed to 60 dB L_den_. While the study was performed in an Alpine valley characterized by very specific noise conditions, other authors studied the association of railway noise with sleep disturbance [4,57]. Furthermore, railway noise is often related to vibrations, which induce other negative effects on sleep [58,59]. In a previous study [60], the authors showed that railway noise maps underestimate noise exposure and people are disturbed by unconventional noises such as brakes, squeals, whistles, and screeches, which are usually not considered in noise modelling. The underestimation of noise and the presence of vibration resulted in an increase of the percentage of highly annoyed people (%HA) with respect to the traditional noise dose–effect curves.

In an urban context, recreational noise plays an important role in citizens’ disturbance, even if it has not yet been well studied yet. In recent years more attention has been paid to the topic [61], but most of studies have only focused on campus students [62]. While the relation of recreational noise to cardiovascular outcomes still needs study, the insurgence of tinnitus, hearing loss and noise-induced hearing-threshold shift due to high levels of music were investigated [63,64] and connections were found by different authors [65]. 

The present study aims to evaluate the impact that different noise sources have on the health of citizens in terms of blood pressure (BP) and hypertension. A sample of 517 citizens living in the city of Pisa, Italy, was chosen for blood pressure measurements and a structured questionnaire. The city of Pisa was a good test site for the study because of its complex structure in terms of noise sources, including all the previously mentioned transportation sources, with an important airport very close to the inhabited areas, and major roads and railway stretches crossing the residential area. The exposure to all of these sound sources was considered as a whole or individually in order to evaluate their eventual correlation with health parameters. In a public health context, the results obtained could be used by institutions and citizens to prevent exposure to specific noise sources.

## 2. Materials and Methods

### 2.1. Sample Selection

The study sample includes 517 subjects, 37–72 years of age at the time of interview, previously selected for the SERA project (study on the effects of airport noise) [66] and the SERA-FA project (study on the effects of airport, railway and recreational noise) [67]. For both projects, the population sample was recruited through a random selection, stratified by gender, age and main sound source from the database of addresses provided by the local General Registry Office. The subjects were extracted uniformly considering sex, age and potential exposure to the principal noise sources according to the noise map of the city. Subsequently, up to three substitutions were selected in order to replace the non-respondents and those who refused to participate.

In the SERA project, the population was recruited in 2012 in a cross-sectional study, with a random sample of adults (45–70 years of age) living in Pisa and exposed to different average noise levels. A first set was exposed to at least 55 dB (A) of airport L_den_, a second was exposed to 50 dB (A) of both airport and traffic L_den_, a third was exposed to at least 55 dB (A) of traffic L_den_ and the last was not exposed to significant noise levels from these main sources. Participants were subjected to blood pressure measurements and to a structured questionnaire using the model adopted in the HYENA study [52]. This included questions on house characteristics, possible protection from noise, windows, socio-demographic conditions, occupational noise exposure, dietary habits, lifestyle factors, smoking, noise annoyance, sleep conditions and noise sensitivity.

From 2014 to 2016 more participants, aged 37–72, were added to the SERA-FA study in order to include subjects exposed to at least 55 dB(A) of railway L_den_ and subjects exposed to at least 55 dB (A) of recreational noise, in terms of L_night._ The same protocol as the SERA project was used, but two sections were added to the questionnaire in order to specifically investigate the exposure to railway and recreational noise.

The questionnaire campaign with the assessment of BP was carried out in 2012–2013 for the SERA project and 2014–2015 for SERA-FA participants.

### 2.2. Exposure Assessment

Noise exposure to the transport infrastructure (road, railway, airport) was obtained by the noise maps developed by the Environment Protection Agency for the Tuscany Region (ARPAT) according to the guidelines of the European Noise Directive 2002/49/EC (END) and the Italian Decree of 2005 (D. Lgs 194/05) [68]. Using the proper input data required by the noise model (i.e., traffic flow, speed), annual average L_den_ and L_night_ of the single source were computed on a grid of 5 m × 5 m positioned at a height of 4 m above the ground, at a distance of 1 m from the building’s façade using the Integrated Noise Model 7.0b (INM) [69]. The overall noise exposure was also calculated as the energetic sum of the three components. These were used to estimate the percentage of residents exposed to noise levels greater than 55 dB (A) L_den_ and 50 dB (A) L_night_. L_den_ and L_night_ were calculated. The German national method VBEB [70] was used as methodology to assign population to noise levels, as a study reported [71] how this better describes real exposure for epidemiological studies, with respect to the method proposed by the END. VBEB distributes the population among the receiver points located around buildings equally, and determines an exposure proportional to noise levels along all the building’s façades, while the END assigns the maximum level from all the points around the corresponding building, which is usually on the most exposed façade [72]. The meteorological parameters considered in the model, such as air temperature, atmospheric pressure, wind speed and relative humidity, were measured by weather stations located in the city of Pisa. Moreover, a measurement campaign for railway noise was conducted in two different parts. In 2013–2014 [73], measurements were performed along the railway lines in 31 places within the city of Pisa with the aim of validating the railway noise map. A class 1 sound level meter, compliant with IEC 61672-1 [74], was placed at a height of 1.5 m and 1 m away from the most exposed façade, recording the A-weighted equivalent continuous sound level (L_Aeq_) with a time step equal to one second. From February to April 2015, the number of measurement points was increased, with another 27 short term and seven measurements providing daily and nightly value for noise exposure. A comparison between noise measurements and the noise map showed that railway infrastructure affects the surrounding areas differently than forecasted, due to the presence of unconventional noise from maneuvering, loading and unloading, truck movements, braking, squeals, whistles, arrivals and departures of trains, speakers, passengers, internal work, generators, bells, crossings, etc. [60]. The resulting differences have been used to correct the citizens’ exposure to railway noise. 

At present, no model can simulate recreational noise, thus a specific measurement campaign which lasted for 18 months was conducted in order to assess the areas within the city of Pisa more subject to this source, such as the city center. Noise data were acquired with the wireless sensor network for real-time noise mapping used in the SENSEable project [75]. L_Aeq_ was acquired simultaneously in six different positions with a temporal base equal to one second, averaged in day-time periods. The measurement points were selected [76] based on the number of residents, in order to optimize the search for similar environments from an acoustic point of view. These are the largest areas possible in which it is possible to assume that the sound pressure level varies within 5 dB (A). The monthly average L_Aeq_ was calculated, eliminating occasional sound events, rain and wind. Recreational noise was defined as that part of noise that exceeded the road traffic noise level resulting from the noise map of the area, as this is the only other noise source affecting the city center. Further details on elaboration and stability can be found in the literature [77]. Estimates were then calculated using the main European indicators (L_den_, L_night_), with standard deviation as a measure of uncertainty, and were assigned to citizens living in similar environments from an acoustic point of view.

Geographical coordinates were assigned to subjects using a common GIS software. For the addresses geocoding, the normalization and georeferencing service of the Tuscany Region has been used. Residents were classified depending on the superposition of noise maps (L_den_ < 55, 55–59, 60–64, 65–69, ≥70).

### 2.3. Assessment of the Outcome

Trained interviewers measured systolic blood pressure (SBP) and diastolic blood pressure (DBP) at subject’s homes after at least five minutes of rest in a seated position keeping both feet on the ground, using an automatic Omron M6 Comfort model (OMRON, Tokyo, Japan) with cuff attached to right or left upper arm (preferably right) [78]. The visits were performed during day-time from Monday to Friday. The staff assessed SBP and DBP three times at each home visit, with the first measurement recorded at the beginning of the interview after 5 min rest, and the second after a further minute, in according with recommendations of the American Heart Association [79]. The third measurement was at the end of the interview, approximately 45 min later. Home visits were distributed over the day in order to account for possible diurnal variations in BP. Two additional measurements were self-made by subjects in the evening of the same day and in the morning of the following day. The average of the 5 measurements provided the SBP and DBP values used in the analysis.

### 2.4. Covariates

An evaluation of the possible major confounders was performed among the variables which can be risk factors for hypertension and possibly associated with noise exposure, in order to eventually exclude them from the model. The potential confounders or effect modifiers that we have evaluated were the usual health indicators (physical activity and body mass index—BMI), sociodemographic characteristics (sex, age, education and employment status), lifestyle habits (smoking and alcohol), other noise sources different than mean noise exposure, work-related noise exposure, noise sensitivity value based on standardized ten questions [80,81] and home conditions (double-glass windows, other noise protections, construction year of the house). Subjects also indicated their annoyance to noise on a 11-point scale for each source on a list of ten: this parameter was evaluated as a potential effect modifier of the investigated relationship.

### 2.5. Statistical Analyses

Standard statistical methods were applied using STATA 14.2 [82]. In addition to SBP and DBP, the prevalence of hypertension based on the self-reported diagnosis was calculated, together with the use of antihypertensive medication or blood pressure measurements reporting SBP ≥ 140 mmHg and DBP ≥ 90 mmHg. This criterion is recommended by the World Health Organization (WHO) [83]. 

Pair-wise correlation between noise map indicators (airport, traffic and railway) were calculated and the association between noise levels and hypertension investigated using a logistic regression model. The odds ratio (OR) and 95% Confidence Intervals (CIs) for each effect estimate were estimated as results of this analysis.

The possible relation between environmental noise levels and BP, expressed as SBP and DBP separately, was assessed with mixed linear regression models and associations expressed with both day-time and night-time noise levels, obtaining risk beta coefficients and 95% CIs.

The analysis in categories made of intervals equal to 5 dB (A) suggested a linear relation, thus continuous exposure data have been used to assess the effect estimate in order to increase statistical power.

Potential covariates were evaluated in non-adjusted analysis: those with a *p* < 0.20 in order to avoid exclusion of important adjustment variables due to stochastic variability [84,85] and those already known in literature as risk factors for hypertension [86] (sex, age, BMI, educational status) were selected. The final model included sex, age (as continuous), educational status (elementary, medium, high school, university), alcohol (never drinker, former drinker and actual drinker), physical activity (less than 1 time a week of moderate exercise, between 1 and 3 times a week, more than 3 times a week), BMI, and use of pre-cooked foods (at least once a week, less than 1 meal at week). Smoking was included only in the model for BP, as a well-known risk factor for heart disease, but not for hypertension, as confirmed by the *p*-value, therefore not relevant in the preliminary analysis.

In order to investigate the differential susceptibility to noise exposure in subgroups of the study population, a stratification of the analysis was performed by sex, age, noise sensitivity (<50th percentile (P50) vs. ≥50th percentile), house noise protection (yes vs. no), windows closed to prevent noise exposure (never, few vs. often, always), living room exposition (noise source vs. side of noise source vs. back of noise source), bedroom exposition (noise source vs. side of noise source vs. back of noise source) and annoyance (few, moderately annoyed vs. very annoyed).

## 3. Results

A total of 517 participants (228 men and 289 women), aged between 35 and 72 years, at the time of visit, participated to the present study. The response rate in the study was medium-low (29.1%). In order to assess the potential selection bias, the authors compared the source population and the sample by sex and age, finding no statistically significant difference. 

The mean age of participants was 57.3 years old (standard deviation 8.7) and 44.1% were males. Mean SBP and DBP expressed in mmHg during the visit were 126.9 and 81.1, respectively, while means for self-measured blood pressure were 125.6/79.2 and 121.5/77.7 respectively, for evening and next day morning. The overall hypertension prevalence was 37.5% (to be compared with the Italian population, in which there is a prevalence of 33% and 31% respectively among males and females [87]); of all subjects, 20.1% were treated for hypertension and had normal values of BP, 11.0% were treated but presented hypertensive values of SBP or DBP, and 11.2% without a medical prescription for hypertension presented abnormal values of BP. The prevalence of hypertension was higher in males (44.6%) than in females (32.7%).

Table 1 describes the variables considered in the study and stratified by hypertensive condition expressed as the WHO classification. Statistically significant differences between the two groups arose. Among those with hypertensive condition, higher values of SBP and DBP, BMI, alcohol consumption, lower level of education, actual workers, less than one time/week of moderate exercise, use of precooked foods and lower attitude to close windows were found.

The mean noise levels of the main noise source considered in this study are 61.7 dB (A) (standard deviation 7.6) of L_den_ and 49.4 dB (A) (standard deviation 13.6) of L_night_.

Multiple associations between covariates and prevalence of hypertension are shown in Table 2. The results represent the relationships between single parameters and risk of hypertension, net of all the other covariates included concurrently, without the main exposure of noise. Variables such as sex (male), higher age, smoking, higher BMI showed significant positive associations with a higher risk of hypertension. Educational level, stability of work conditions and physical activity showed a protective effect, in a significant association with a lower risk of hypertension.

Table 3 shows correlation coefficients between environmental noise exposure levels by day and night. Values display a different correlation for each noise source (r = 0.22 for airport noise, 0.99 for both railway and traffic exposures). In addition, significant correlation values between airport noise and railway noise during nighttime were detected, whilst for railways, this seems to be at the boundary of significance during daytime.

The regression model shown in Table 4, indicates that a 5 dB (A) increase in nocturnal environmental noise corresponds to a significant increase in blood pressure, especially in DBP (DBP and night-time noise: β = 0.50, 95% confidence intervals (CIs): 0.18, 0.81). Considering the hypertensive outcome, associations are almost significant especially during night-time in the full adjusted model (OR = 1.07, 95% CI: 0.99–1.15). Night-time noise is involved too in the association with SBP, showing a nearly significant association (β = 0.47, 95% CI: −0.05, 1.00).

Stratifying the main characteristics, the effects estimates were higher in participants who showed a higher noise sensitivity (based on Weinstein’s noise sensitivity method). Association between hypertension and environmental nocturnal noise were found in males (OR = 1.13; 95% CI: 1.01, 1.26), in persons older than 65 years of age (OR = 1.18; 95% CI: 1.02, 1.37) and those with higher noise sensitivity (OR = 1.12; 95% CI: 1.01–1.24).

**Figure 1:**
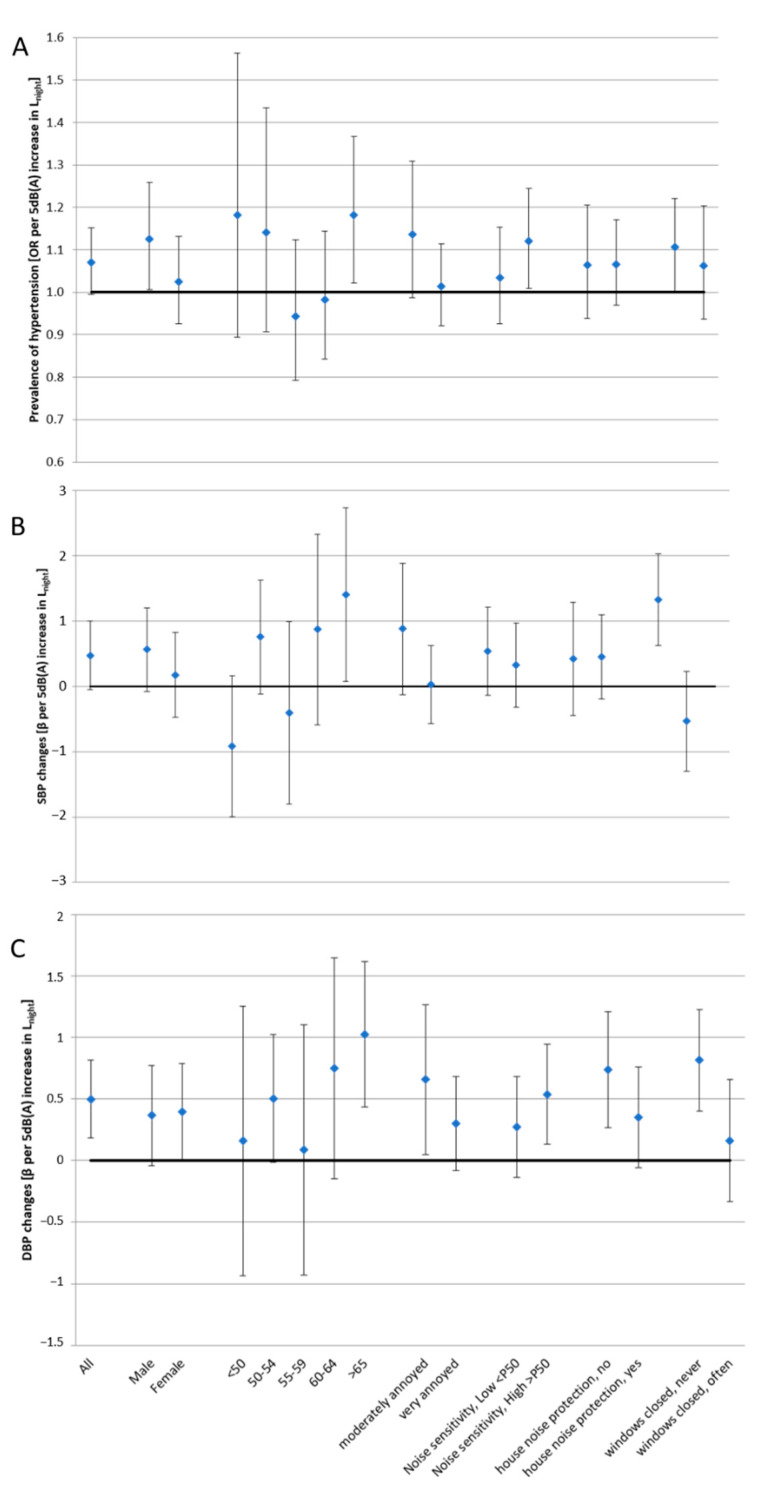
Estimated ORs/β per increment of 5 dB(A) of night-time noise by subgroups of population. in prevalent hypertension (**A**), estimated change of SBP (**B**) and estimated changes of DBP (**C**).

Relations between DBP and environmental nocturnal noise showed some significant results too, among all participants, females (β = 0.40; 95% CI: 0.00, 0.79), people aged over 65 years (β = 1.03; 95% CI: 0.43, 1.62), people moderately annoyed by noise (β = 0.66; 95% CI: 0.05, 1.27) and other categories, shown in Figure 1, as noise sensitivity below and above the 50th percentile (45 in a scale from 10 to 60), structural changes for house noise protection (yes vs. no) and the habit of closing windows (never, few vs. often, always).

**Figure 2:**
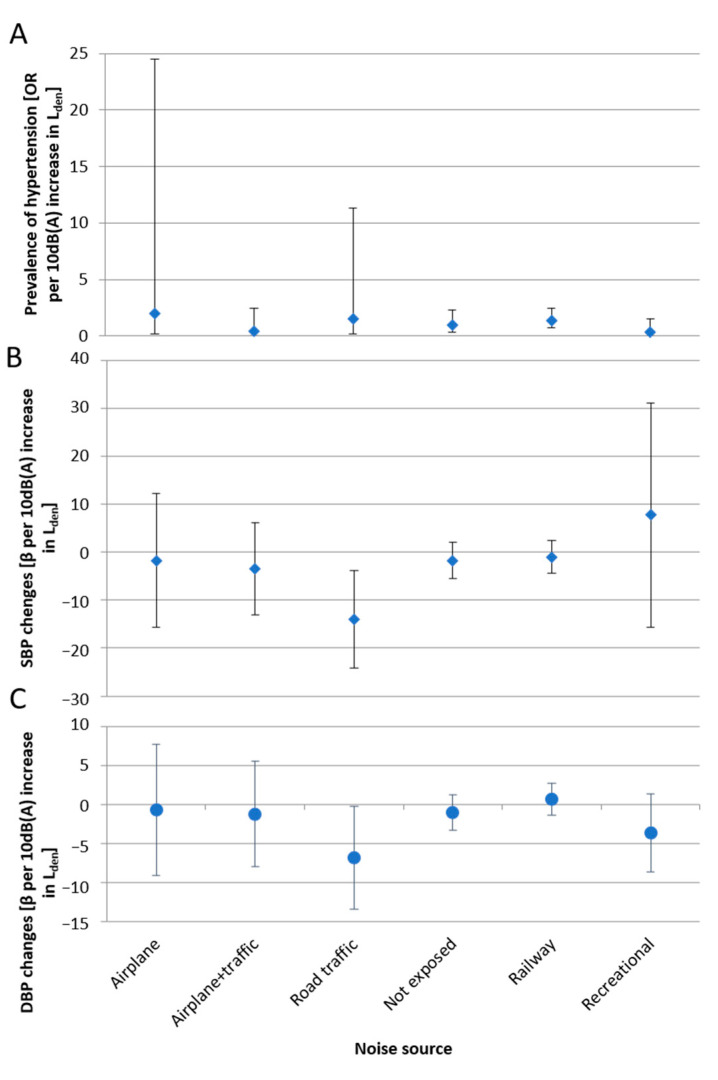
Estimated ORs/β per increment of 5 dB(A) of noise by main noise exposure. in prevalent hypertension (**A**), estimated change of SBP (**B**) and estimated changes of DBP (**C**).

Noise could be quite different in terms of frequency, amplitude and duration of exposure. Figure 2 reports β for an increment of 10 dB(A) in L_den_, stratified by main noise exposure: only railway exposure has a positive value (β = 0.68; 95% CI: −1.36, 2.72), although it does not reach a statistically significant level of risk.

## 4. Discussion

The present study investigated the impact of exposure to multiple noise sources on the blood pressure and on onset of hypertension in the wake of the HYENA study. Significant positive exposure–response relationships, especially to night-time noise exposure, were found in males; people aged over 65 years old and with a high sensitivity to noise in association with hypertension,; people aged over 65 years old and who never close windows at home due to noise in association with increase in systolic blood pressure; and for all participants, females, people aged over 65 years old, moderately annoyed by noise, high sensitivity to noise, without house noise protection and who never close windows due to noise, in association with an increase in diastolic blood pressure.

Significant differences were found in the hypertensive outcome between the various noise sources, considered to be road, railway and airport traffics and recreational noise. The exposure-response relationships between sound levels and cardiovascular outcomes showed different ORs depending on the sound sources analyzed; railway noise showed highest ORs. It should be noted that railway and road traffic noise were highly correlated between day and night, unlike aircraft noise. A possible explanation is that aircraft activity in Pisa is limited during the night. Nevertheless, the L_night_ indicator (10 p.m.–6 a.m.) includes the so-called “shoulder hours” of the late evening and early morning, where some planes fly in an environment with a background noise lower than that in the day-time.

It is reasonable to believe that the relationship highlighted between nocturnal noise and hypertension can be motivated by the fact that the participants spent the night inside their houses compared to daytime hours, since the noise level assigned to the home address was used. This procedure would also explain the lower misclassification exposure during night hours, compared to daytime. Indeed, it is therefore reasonable to assume that during night hours the participants were actually subjected to the sound levels shown and that therefore there may be a correlation with cardiovascular effects, as emerged from the analyzes [88].

Smoking and alcohol are historical risk factors for hypertension, although smoking is still under investigation for its effect on blood pressure. For this reason, subjects were asked to refrain from smoking during the 30 min before the interview and BP measurement. As detailed in the methods section, smoking was included as a variable in the model, even if its impact on estimate of the exposure–response was not relevant. 

The exposure–response association for hypertensive risk was more relevant among men, in accordance with previous evidence on males and hypertension [89,90,91]. However, the studies mentioned only investigated the relation between road traffic noise and hypertension. The present study aimed to consider a larger number of noise sources in a city like Pisa, where citizens are often exposed to a mixture of noise pollutants. Even when transportation noise seems to be absent, such as in the city center, anthropogenic noise could play a role among the determinants of cardiovascular and sleep health.

In all the investigated outcomes (hypertension, SBP and DBP), variable “age” gave the same indication of higher risk for people aged over 65 years, given that this category is likely to spend more time at home, and consequently its exposure should be less commonly misclassified. 

Age always shows positive values and reaches significance in all night-noise analysis for 5 dB(A) increase in L_night_ (Hypertension OR = 1.18; 95% CI: 1.02–1.37; SBP β = 1.41; 95% CI: 0.08–2.73; DBP β = 1.03; 95% CI: 0.43–1.62).

Gathering together some of the subcategories detected in the questionnaire, as reported in Figure 1, it emerged that, in the relation between DPB increase and L_night_ noise, significant risks were found in subjects moderately annoyed ((0–7 in a 11-point scale) β = 0.66; 95% CI, 0.05–1.27), or with lower noise sensitivity, beyond 50th percentile, (β = 0.54; 95% CI, 0.13–0.94), or living in a house free of noise protection (β = 0.74; 95% CI, 0.26–1.21) or who never close windows because of noise (β = 0.82; 95% CI, 0.40–1.23). Apparently, people who not protecting themselves from noisy sources for personal reasons are more at risk than those who, concerned about the possible effects of noise on their health, strives to protect themselves from this specific exposure.

A potential weakness of this study is the medium-low response rate. However, a descriptive analysis showed that response rate is not different by sex, age and exposure zones, the only exception being the aircraft noise group showing a higher response rate. This can be partially explained by taking into account the limited population of the city (almost 90,000 residents) with a high component of students, and the airport, which is very close to the city, represents the major environmental concern of citizens.

Another weakness of the study could be the different exposure assessment of recreational noise, involving no initial “pedestrian data flow”, several microphones in specific areas and a subsequent model. A misclassification and a problem of comparison between noise sources could exist, as no data on recreational noise outside of the city center of Pisa were available. On these bases, the recreational group resulted in a non-significant and negative relation in all analyses, therefore the results, including all areas, could be underestimates.

At the same time, the present study focused not on a single type of noise, but shifted attention towards a more comprehensive approach to noise exposure that involves citizens in several ways, each with its peculiarities (frequency content, amplitude, individual perception, etc.). In addition, the completeness of the questionnaire helped to clarify certain factors and their roles in the associations investigated.

## 5. Conclusions

Statistically significant positive relation between night-time noise and diastolic blood pressure was found. The subcategories majorly involved in the relation between night-time noise and diastolic blood pressure were people aged older than 65 years, moderately annoyed, noise sensitive, without noise protections in house and residents who usually do not close windows when exposed to high levels of noise. Among various noise sources, railway noise showed the strongest association with the outcomes of the study. Hypertension is a major independent risk factor for events such as myocardial infarction and stroke and this study demonstrated an increase of risk in association with environmental noise.

## Figures and Tables

**Table 1:** Characteristics of subjects included in the study, variables divided in continuous and categorical variables.

Characteristics	Total (n = 515)	Non-Hypertensive (n = 313)	Hypertensive (n = 194)	*p*-Value ^a^
Continuous variables [median (IQR)]				
Systolic blood pressure (mmHg)	123.1 (20.0)	117.5 (16.0)	136.5 (20.5)	<0.001 *
Diastolic blood pressure (mmHg)	78.5 (12.1)	76.0 (9.25)	86.8 (13.25)	<0.001 *
Age (years)	58.2 (14.2)	54.8 (12.8)	62.7 (11.3)	<0.001 *
Main noise source L_DEN_ [dB(A)]	62 (10.0)	61.6 (10.6)	62.5 (10.2)	0.066
Main noise source L_NIGHT_ [dB(A)]	53.5 (18.0)	53.1 (15.4)	54.1 (18.3)	0.254
Noise sensitivity score (10–60) ^b^	39.0 (12.0)	39.0 (12.0)	39.0 (11.0)	0.874
Categorical variables [n (%)]				
Male sex	228 (44.1)	124 (39.6)	101 (50.2)	0.042
BMI (Kg/m^2^)				<0.001 *
<18.5	9 (1.7)	8 (2.6)	1 (0.5)	
18.5–24.9	251 (48.6)	178 (56.9)	73 (36.3)	
25–29.9	201 (38.9)	97 (31.0)	103 (51.2)	
30+	56 (10.8)	30 (9.6)	24 (11.9)	
Educational level				<0.001 *
University or similar	241 (46.6)	166 (53.0)	73 (36.3)	
Secondary	174 (33.7)	103 (32.9)	70 (34.8)	
Primary	66 (12.8)	31 (9.9)	35 (17.4)	
Illiterate	33 (6.4)	12 (3.8)	21 (10.5)	
Smoking				0.501
Professional Status				
Unemployed	58 (11.5)	34 (10.9)	24 (12.5)	
Retired	157 (31.1)	78 (24.9)	79 (24.9)	
Actual worker	290 (64.6)	201 (64.2)	89 (64.2)	<0.001 *
Physical activity (moderate exercise)				
Less than 1 time/week	45 (8.9)	20 (6.4)	25 (13.0)	
Between 1 and 3 times/week	134 (26.5)	91 (29.2)	43 (22.4)	
More than 3 times/week	326 (64.6)	202 (64.5)	124 (64.6)	0.020
Never smokers	235 (45.5)	148 (47.3)	85 (42.3)	
Smokers	116 (22.4)	72 (23.0)	44 (21.9)	
Former smokers	166 (32.1)	93 (29.7)	72 (35.8)	
Drinking ^c^				0.009 *
Non-drinkers	146 (28.2)	98 (31.3)	48 (23.9)	
Casual drinkers	187 (38.2)	117 (37.4)	68 (33.8)	
Regular drinkers	183 (35.4)	98 (31.3)	84 (41.8)	
Diet, use of pre-cooked foods	208 (40.2)	142 (45.4)	64 (31.8)	0.009 *
Living room orientation, noise source ^d^	128 (24.8)	77 (24.6)	51 (25.4)	0.882
Bedroom orientation, noise source ^d^	134 (25.9)	84 (26.8)	48 (23.9)	0.571
Closing windows, yes ^e^	162 (31.3)	107 (34.2)	54 (26.9)	0.081
Protections, yes ^f^	333 (64.4)	207 (66.1)	124 (61.7)	0.395
Noise annoyance ^g^				0.878
Moderate (0–7)	201 (39.0)	119 (45.9)	75 (45.2)	
High (8–10)	231 (44.9)	140 (54.1)	91 (54.8)	
Not exposed	83 (16.1)	54 (67.5)	26 (32.5)	
Air pollution annoyance, high ^h^	281 (54.4)	170 (54.3)	110 (54.7)	0.200
Noise groups				0.018
Aircraft	100 (19.8)	59 (18.9)	41 (21.4)	
Road traffic + Aircraft	80 (15.8)	40 (12.8)	40 (20.8)	
Road traffic	74 (14.7)	42 (13.4)	32 (16.7)	
Railway	118 (23.4)	85 (27.2)	33 (17.2)	
Recreational	53 (10.5)	33 (10.5)	20 (10.4)	
Reference group	80 (15.8)	54 (17.3)	26 (13.5)	

^a^ Chi-square test and Kruskall-Wallis test for strata of hypertension with categorical or continuous variables, respectively. ^b^ Higher noise sensitivity with higher values. ^c^ Casual: less than 2 glasses/week. (1 missing observation) ^d^ Control group not included. ^e^ Yes: always close windows (vs. no: never, only on summer or winter season). ^f^ Sound-proofed windows or changes in structure due to noise. ^g^ Referred to the main noise source; in case of aircraft/traffic group, the higher annoyance is selected. ^h^ Score from 6 to 10 in a 0–10 scale. * Category with a significant association with hypertension.

**Table 2:** Multiple associations between covariates and the prevalence of hypertension (HYENA definition).

Variable	Categorization	OR ^a^ (95% CI)	*p*-Value
Age	Per 1 year	1.08 (1.05–1.10)	<0.001
Gender	Male	1	
	Women	0.70 (0.46–1.08)	0.107
Alcohol	Never drinker	1	
	Casual drinker	1.25 (0.75–2.10)	0.394
	Regular drinker	1.30 (0.76–2.20)	0.338
Smoking	Never smoker	1	
	Former smoker	0.79 (0.49–1.26)	0.321
	Actual smoker	0.69 (0.41–1.17)	0.169
Professional status	Unemployed	1	
	Retired	0.38 (0.19–0.75)	0.006
	Actual worker	0.49 (0.26–0.92)	0.027
BMI	Per kg/m^2^	1.07 (1.02–1.13)	0.010
Noise sensitivity	Per scale unit	1.01 (0.99–1.03)	0.225
Educational level	Illiterate	1	
	Primary	0.91 (0.36–2.32)	0.844
	Secondary	0.61 (0.26–1.41)	0.284
	University or similar	0.47 (0.20–1.09)	0.117
Physical activity	None	1	
	Moderately or strenuous 1–3 times a week	0.43 (0.20–0.91)	0.028
	Moderately or strenuous > 3 times a week	0.57 (0.28–1.13)	0.108

^a^ Odds Ratio are mutually adjusted.

**Table 3:** Descriptive statistics and results of Spearman’s correlation of the different environmental noise exposures.

	Mean ± SE	Percentile			Air Traffic		Railway		Road Traffic	
		10th	50th	90th	Day	Night	Day	Night	Day	Night
Air Traffic (day)	57.00 ± 0.20	54.5	57.4	59.3	1					
Air traffic (night)	27.78 ± 0.87	20	25.6	41.1	0.22	1				
Railway (day)	59.53 ± 0.78	46.2	61	70.3	−0.12	0.24	1			
Railway (night)	52.49 ± 0.78	39.2	54.3	63.1	−0.12	0.25	0.99 *	1		
Traffic (day)	68.04 ± 0.36	63.9	68	72	0.05	0.00	−0.07	−0.07	1	
Traffic (night)	59.15 ± 0.37	55.3	59.2	63.4	0.05	0.02	−0.06	−0.06	0.99 *	1
Recreational (day) ^a^	70.03 ± 0.66	64.2	71.2	74.2						
Recreational (night) ^a^	63.80 ± 0.72	57.5	65.6	68.4						

^a^ Recreational noise information are missing for the other types of noise. * Significant value.

**Table 4:** Associations between hypertension and blood pressure with environmental noise by day and night; estimated risk for hypertension and change in blood pressure (mmHg) for a 10 dB (A) increment during the day or for a 5 dB (A) increment during night.

Outcome		Night		Day	
		**OR/5 dB(A) (95% CI)**	***p*** **-Value**	**OR/10 dB(A) (95% CI)**	***p*** **-Value**
Hypertension	Non-adjusted	1.03 (0.96, 1.10)	0.386	1.23 (0.97, 1.57)	0.091
	Full model	1.07 (0.99, 1.15)	0.070	1.27 (0.97, 1.67)	0.085
		**β/5 dB (A) (95% CI)**		**β/10 dB (A) (95% CI)**	
SBP	Non-adjusted	0.11 (−0.47, 0.69)	0.715	−0.31 (−2.38, 1.76)	0.768
	Full model	0.47 (−0.05, 1.00)	0.078	−0.08 (−1.97, 1.81)	0.934
DBP	Non-adjusted	0.28 (−0.05, 0.61)	0.101	0.30 (−0.88, 1.48)	0.615
	Full model	0.50 (0.18, 0.81)	0.002	0.91 (−0.23, 2.06)	0.118

## Data Availability

The data presented in this study are available on request from the corresponding author.

## References

[1] Kujawa S.G., Liberman M.C. (2006). Acceleration of age-related hearing loss by early noise exposure: Evidence of a misspent youth. J. Neurosci..

[2] Sliwinska-Kowalska M., Davis A. (2012). Noise-induced hearing loss. Noise Health.

[3] Miedema H.M.E., Vos H. (2007). Associations between self-reported sleep disturbance and environmental noise based on reanalyses of pooled data from 24 studies. Behav. Sleep Med..

[4] Basner M., McGuire S. (2018). WHO environmental noise guidelines for the european region: A systematic review on environmental noise and effects on sleep. Int. J. Environ. Res. Public Health.

[5] Miedema H.M., Oudshoorn C.G. (2001). Annoyance from transportation noise: Relationships with exposure metrics DNL and DENL and their confidence intervals. Environ. Health Perspect..

[6] Eze I.C., Foraster M., Schaffner E., Vienneau D., Héritier H., Pieren R., Thiesse L., Rudzik F., Rothe T., Pons M. (2018). Transportation noise exposure, noise annoyance and respiratory health in adults: A repeated-measures study. Environ. Int..

[7] Sung J.H., Lee J., Jeong K.S., Lee S., Lee C., Jo M.-W., Sim C.S. (2017). Influence of Transportation Noise and Noise Sensitivity on Annoyance: A Cross-Sectional Study in South Korea. Int. J. Environ. Res. Public Health.

[8] Guski R., Schreckenberg D., Schuemer R. (2017). WHO environmental noise guidelines for the European region: A systematic review on environmental noise and annoyance. Int. J. Environ. Res. Public Health.

[9] Muzet A. (2007). Environmental noise, sleep and health. Sleep Med. Rev..

[10] Park T., Kim M., Jang C., Choung T., Sim K.-A., Seo D., Chang S.I. (2018). The Public Health Impact of Road-Traffic Noise in a Highly-Populated City, Republic of Korea: Annoyance and Sleep Disturbance. Sustainability.

[11] Hygge S., Evans G.W., Bullinger M. (2002). A prospective study of some effects of aircraft noise on cognitive performance in schoolchildren. Psychol. Sci..

[12] Lercher P., Evans G.W., Meis M. (2003). Ambient Noise and Cognitive Processes among Primary Schoolchildren. Environ. Behav..

[13] Clark C., Paunovic K. (2018). WHO environmental noise guidelines for the european region: A systematic review on environmental noise and cognition. Int. J. Environ. Res. Public Health.

[14] Daiber A., Kröller-Schön S., Frenis K., Oelze M., Kalinovic S., Vujacic-Mirski K., Kuntic M., Bayo Jimenez M.T., Helmstädter J., Steven S. (2019). Environmental noise induces the release of stress hormones and inflammatory signaling molecules leading to oxidative stress and vascular dysfunction—Signatures of the internal exposome. Biofactors.

[15] Basner M., Babisch W., Davis A., Brink M., Clark C., Janssen S., Stansfeld S. (2014). Auditory and non-auditory effects of noise on health. Lancet.

[16] Münzel T., Gori T., Babisch W., Basner M. (2014). Cardiovascular effects of environmental noise exposure. Eur. Heart J..

[17] Dratva J., Phuleria H.C., Foraster M., Gaspoz J.-M., Keidel D., Künzli N., Liu L.-J.S., Pons M., Zemp E., Gerbase M.W. (2012). Transportation noise and blood pressure in a population-based sample of adults. Environ. Health Perspect..

[18] Van Kempen E., Casas M., Pershagen G., Foraster M. (2018). WHO environmental noise guidelines for the European region: A systematic review on environmental noise and cardiovascular and metabolic effects: A summary. Int. J. Environ. Res. Public Health.

[19] Vukić L., Mihanović V., Fredianelli L., Plazibat V. (2021). Seafarers’ Perception and Attitudes towards Noise Emission on Board Ships. Int. J. Environ. Res. Public Health.

[20] Themann C.L., Masterson E.A. (2019). Occupational noise exposure: A review of its effects, epidemiology, and impact with recommendations for reducing its burden. J. Acoust. Soc. Am..

[21] Caviola S., Visentin C., Borella E., Mammarella I., Prodi N. (2021). Out of the noise: Effects of sound environment on maths performance in middle-school students. J. Environ. Psychol..

[22] Berglund B., Lindvall T., Schwela D.H. (2000). New Who Guidelines for Community Noise. Noise Vib. Worldw..

[23] Lim S.S., Vos T., Flaxman A.D., Danaei G., Shibuya K., Adair-Rohani H., Amann M., Anderson H.R., Andrews K.G., Aryee M. (2012). A comparative risk assessment of burden of disease and injury attributable to 67 risk factors and risk factor clusters in 21 regions, 1990–2010: A systematic analysis for the Global Burden of Disease Study 2010. Lancet.

[24] Van Kempen E., Babisch W. (2012). The quantitative relationship between road traffic noise and hypertension: A meta-analysis. J. Hypertens..

[25] Ward B.W., Schiller J.S. (2013). Prevalence of multiple chronic conditions among US adults: Estimates from the National Health Interview Survey, 2010. Prev. Chronic Dis..

[26] Ward B.W., Schiller J.S., Goodman R.A. (2014). Multiple chronic conditions among US adults: A 2012 update. Prev. Chronic Dis..

[27] Banerjee D., Das P.P., Fouzdar A. (2014). Urban residential road traffic noise and hypertension: A cross-sectional study of adult population. J. Urban Health.

[28] Floud S., Blangiardo M., Clark C., de Hoogh K., Babisch W., Houthuijs D., Swart W., Pershagen G., Katsouyanni K., Velonakis M. (2013). Exposure to aircraft and road traffic noise and associations with heart disease and stroke in six European countries: A cross-sectional study. Environ. Health.

[29] Haralabidis A.S., Dimakopoulou K., Velonaki V., Barbaglia G., Mussin M., Giampaolo M., Selander J., Pershagen G., Dudley M.-L., Babisch W. (2011). Can exposure to noise affect the 24 h blood pressure profile? Results from the HYENA study. J. Epidemiol. Community Health.

[30] Kearney P.M., Whelton M., Reynolds K., Muntner P., Whelton P.K., He J. (2005). Global burden of hypertension: Analysis of worldwide data. Lancet.

[31] Babisch W., Fromme H., Beyer A., Ising H. (2001). Increased catecholamine levels in urine in subjects exposed to road traffic noise: The role of stress hormones in noise research. Environ. Int..

[32] Babisch W. (2003). Stress hormones in the research on cardiovascular effects of noise. Noise Health.

[33] Babisch W. (2011). Cardiovascular effects of noise. Noise Health.

[34] Hammer M.S., Swinburn T.K., Neitzel R.L. (2014). Environmental noise pollution in the United States: Developing an effective public health response. Environ. Health Perspect..

[35] European Commission (2017). European Commission Report from the Commission to the European Parliament and the Council on the Implementation of the Environmental Noise Directive in Accordance with Article 11 of Directive 2002/49/EC. COM/2017/0151 Final.

[36] European Environment Agency (EEA) (2014). Noise in Europe 2014.

[37] Morley D.W., de Hoogh K., Fecht D., Fabbri F., Bell M., Goodman P.S., Elliott P., Hodgson S., Hansell A.L., Gulliver J. (2015). International scale implementation of the CNOSSOS-EU road traffic noise prediction model for epidemiological studies. Environ. Pollut..

[38] Ruiz-Padillo A., Ruiz D., Torija A., Ramos-Ridao Á. (2016). Selection of suitable alternatives to reduce the environmental impact of road traffic noise using a fuzzy multi-criteria decision model. Environ. Impact Assess. Rev..

[39] Bunn F., Zannin P.H.T. (2016). Assessment of railway noise in an urban setting. Appl. Acoust..

[40] Gagliardi P., Fredianelli L., Simonetti D., Licitra G. (2017). ADS-B system as a useful tool for testing and redrawing noise management strategies at Pisa Airport. Acta Acust. United Acust..

[41] Iglesias Merchan C., Diaz-Balteiro L., Soliño M. (2015). Transportation planning and quiet natural areas preservation: Aircraft overflights noise assessment in a National Park. Transp. Res. Part D Transp. Environ..

[42] Kephalopoulos S., Paviotti M., Anfosso-Lédée F., Van Maercke D., Shilton S., Jones N. (2014). Advances in the development of common noise assessment methods in Europe: The CNOSSOS-EU framework for strategic environmental noise mapping. Sci. Total Environ..

[43] Morel J., Marquis-Favre C., Gille L.-A. (2016). Noise annoyance assessment of various urban road vehicle pass-by noises in isolation and combined with industrial noise: A laboratory study. Appl. Acoust..

[44] Borelli D. (2019). Maritime Airborne Noise: Ships and Harbours. Int. J. Acoust. Vib..

[45] Fredianelli L., Bolognese M., Fidecaro F., Licitra G. (2021). Classification of Noise Sources for Port Area Noise Mapping. Environments.

[46] Eriksson C., Rosenlund M., Pershagen G., Hilding A., Ostenson C.-G., Bluhm G. (2007). Aircraft noise and incidence of hypertension. Epidemiology.

[47] Huss A., Spoerri A., Egger M., Röösli M. (2010). Aircraft noise, air pollution, and mortality from myocardial infarction. Epidemiology.

[48] Licitra G., Gagliardi P., Fredianelli L., Simonetti D. (2014). Noise mitigation action plan of Pisa civil and military airport and its effects on people exposure. Appl. Acoust..

[49] Basner M., Müller U., Elmenhorst E.-M. (2011). Single and combined effects of air, road, and rail traffic noise on sleep and recuperation. Sleep.

[50] Fritschi L., Brown L., Kim R., Schwela D.H., Kephalopoulos S. (2011). Burden of Disease from Environmental Noise. Quantification of Healthy Life Years Lost in Europe.

[51] Hellmuth T., Classen T., Kim R., Kephalopoulos S. (2012). Methodological Guidance for Estimating the Burden of Disease from Environmental Noise.

[52] Jarup L., Babisch W., Houthuijs D., Pershagen G., Katsouyanni K., Cadum E., Dudley M.-L., Savigny P., Seiffert I., Swart W. (2008). Hypertension and exposure to noise near airports: The HYENA study. Environ. Health Perspect..

[53] Eriksson C., Bluhm G., Hilding A., Ostenson C.-G., Pershagen G. (2010). Aircraft noise and incidence of hypertension—Gender specific effects. Environ. Res..

[54] Rosenlund M., Berglind N., Pershagen G., Järup L., Bluhm G. (2001). Increased prevalence of hypertension in a population exposed to aircraft noise. Occup. Environ. Med..

[55] Lercher P., Brink M., Rudisser J., Van Renterghem T., Botteldooren D., Baulac M., Defrance J. (2010). The effects of railway noise on sleep medication intake: Results from the ALPNAP-study. Noise Health.

[56] Lercher P., Botteldooren D., Widmann U., Uhrner U., Kammeringer E. (2011). Cardiovascular effects of environmental noise: Research in Austria. Noise Health.

[57] Miedema H.M.E., Vos H. (1998). Exposure response functions for transportation noise. J. Acoust. Soc. Am..

[58] Persson Waye K., Bengtsson J., Agge A., Björkman M. (2003). A descriptive cross-sectional study of annoyance from low frequency noise installations in an urban environment. Noise Health.

[59] Smith M.G., Croy I., Ogren M., Persson Waye K. (2013). On the influence of freight trains on humans: A laboratory investigation of the impact of nocturnal low frequency vibration and noise on sleep and heart rate. PLoS ONE.

[60] Licitra G., Fredianelli L., Petri D., Vigotti M.A. (2016). Annoyance evaluation due to overall railway noise and vibration in Pisa urban areas. Sci. Total Environ..

[61] Ottoz E., Rizzi L., Nastasi F. (2018). Recreational noise: Impact and costs for annoyed residents in Milan and Turin. Appl. Acoust..

[62] Le Prell C.G., Siburt H.W., Lobarinas E., Griffiths S.K., Spankovich C. (2018). No Reliable Association Between Recreational Noise Exposure and Threshold Sensitivity, Distortion Product Otoacoustic Emission Amplitude, or Word-in-Noise Performance in a College Student Population. Ear Hear..

[63] Henderson E., Testa M.A., Hartnick C. (2011). Prevalence of noise-induced hearing-threshold shifts and hearing loss among US youths. Pediatrics.

[64] Serra M.R., Biassoni E.C., Richter U., Minoldo G., Franco G., Abraham S., Carignani J.A., Joekes S., Yacci M.R. (2005). Recreational noise exposure and its effects on the hearing of adolescents. Part I: An interdisciplinary long-term study. Int. J. Audiol..

[65] Śliwińska-Kowalska M., Zaborowski K. (2017). WHO Environmental Noise Guidelines for the European Region: A Systematic Review on Environmental Noise and Permanent Hearing Loss and Tinnitus. Int. J. Environ. Res. Public Health.

[66] Ancona C., Golini M.N., Mataloni F., Camerino D., Chiusolo M., Licitra G., Ottino M., Pisani S., Cestari L., Vigotti M.A. (2014). Health Impact Assessment of airport noise on people living nearby six Italian airports. Epidemiol. Prev..

[67] Vigotti M.A., Petri D., Fredianelli L., Licitra G., Ancona C. Railway noise and blood pressure levels in Pisa Population, Italy. Proceedings of the International Symposium of Engineering Education.

[68] Panicucci A. (2008). Definizione Della Mappatura Acustica Strategica del Comune di Pisa ai Sensi della Direttiva Europea 49/2002/EC. Ph.D. Thesis.

[69] He H., Dinges E., Hermann J., Rickel D., Mirsky L., Roof C., Boeker E., Gerbi P., Senzig D.A. (2007). Integrated Noise Model (INM) Version 7.0 User’s Guide.

[70] Umwelt Bundes Amt Vorläufige Berechnungsmethode zur Ermittlung der Belastetenzahlen durch Umge-Bungslärm—VBEB: 2007. https://www.umweltbundesamt.de/sites/default/files/medien/pdfs/VBEB.pdf.

[71] Licitra G., Ascari E., Brambilla G. Comparative analysis of methods to evaluate noise exposure and annoyance of people. Proceedings of the 20th International Congress on Acoustics 2010, ICA 2010—Incorporating Proceedings of the 2010 Annual Conference of the Australian Acoustical Society.

[72] Licitra G., Ascari E., Fredianelli L. (2017). Prioritizing Process in Action Plans: A Review of Approaches. Curr. Pollut. Rep..

[73] Fredianelli L., Petri D., Licitra G., Vigotti M.A. Railways Noise Assessment in Urban Area for Evaluating Health Effect. Proceedings of the 7th Forum Acusticum.

[74] International Electrotechnical Commission (2013). Electroacoustics—Sound Level Meters—Part 1: Specifications (IEC 61672-1).

[75] Nencini L., De Rosa P., Ascari E., Vinci B., Alexeeva N. SENSEable Pisa: A wireless sensor network for real-time noise mapping. Proceedings of the European Conference on Noise Control.

[76] Nencini L., Vinci B., Vigotti M.A. Setup della rete SENSEABLE Pisa per la realizzazione di uno studio di valuazione degli effetti del rumore antropico sulla salute dei cittadini. Proceedings of the 41 Convegno Nazionale Associazione Italiana di Acustica.

[77] Sotirakopoulos K., Barham R., Piper B., Nencini L. (2015). A statistical method for assessing network stability using the Chow test. Environ. Sci. Process. Impacts.

[78] Ackermann-Liebrich U., Kuna-Dibbert B., Probst-Hensch N.M., Schindler C., Dietrich D.F., Stutz E.Z., Bayer-Oglesby L., Baum F., Brändli O., Brutsche M. (2005). Follow-up of the Swiss Cohort Study on Air Pollution and Lung Diseases in Adults (SAPALDIA 2) 1991-2003: Methods and characterization of participants. Soz. Praventivmed..

[79] Pickering T.G., Hall J.E., Appel L.J., Falkner B.E., Graves J., Hill M.N., Jones D.W., Kurtz T., Sheps S.G., Roccella E.J. (2005). Recommendations for blood pressure measurement in humans and experimental animals: Part 1: Blood pressure measurement in humans: A statement for professionals from the Subcommittee of Professional and Public Education of the American Heart Association Co. Circulation.

[80] Luz G. (2011). A tutorial on noise-sensitivity. J. Acoust. Soc. Am..

[81] Weinstein N.D. (1978). Individual differences in reactions to noise: A longitudinal study in a college dormitory. J. Appl. Psychol..

[82] StataCorp (2015). Stata Statistical Software: Release 14.

[83] Chalmers J., MacMahon S., Mancia G., Whitworth J., Beilin L., Hansson L., Neal B., Rodgers A., Ni Mhurchu C., Clark T. (1999). 1999 World Health Organization-International Society of Hypertension Guidelines for the management of hypertension. Guidelines sub-committee of the World Health Organization. Clin. Exp. Hypertens..

[84] Heinze G., Dunkler D. (2017). Five myths about variable selection. Transpl. Int..

[85] Grant S.W., Hickey G.L., Head S.J. (2019). Statistical primer: Multivariable regression considerations and pitfalls. Eur. J. Cardio-Thorac. Surg..

[86] World Health Organization (2020). HEARTS Technical Package.

[87] Giampaoli S., Vescio M.F., Gaggioli A., Vanuzzo D. (2002). Prevalenza dell’ipertensione arteriosa nella popolazione Italiana. Bollettino Epidemiol. Naz..

[88] Davies R.J., Belt P.J., Roberts S.J., Ali N.J., Stradling J.R. (1993). Arterial blood pressure responses to graded transient arousal from sleep in normal humans. J. Appl. Physiol..

[89] Babisch W., Beule B., Schust M., Kersten N., Ising H. (2005). Traffic noise and risk of myocardial infarction. Epidemiology.

[90] Belojevic G., Saric-Tanaskovic M. (2002). Prevalence of Arterial Hypertension and Myocardial Infarction in Relation to Subjective Ratings of Traffic Noise Exposure. Noise Health.

[91] Herbold M., Hense H.W., Keil U. (1989). Effects of road traffic noise on prevalence of hypertension in men: Results of the Luebeck Blood Pressure Study. Soz. Praventivmed..

